# Association of Common Medications and the Risk of Early-Onset Gastric Cancer: A Population-Based Matched Study

**DOI:** 10.1155/2021/2670502

**Published:** 2021-12-02

**Authors:** Taleen A. MacArthur, William Scott Harmsen, Jay Mandrekar, Feven Abraha, Travis E. Grotz

**Affiliations:** ^1^Department of Surgery, Mayo Clinic, 200 1st Street SW, Rochester, MN 55905, USA; ^2^Department of Biostatistics, Mayo Clinic, 200 1st Street SWS, Rochester, MN 55905, USA

## Abstract

**Background:**

Early-onset gastric cancer (EOGC, age ≤ 60 years at diagnosis) now comprises >30% of new gastric cancers in the United States. It is hypothesized that chronic acid suppression with proton-pump inhibitors (PPIs) may promote tumorigenesis, while other medications including statins, nonsteroidal anti-inflammatory drugs (NSAIDs), metformin, and cyclooxygenase-2 (COX-2) inhibitors have been proposed as protective. We aimed to assess for an association between use of the aforementioned commonly prescribed medications and EOGC development.

**Methods:**

We used a population-based medical record linkage system, to identify cases of EOGC in Olmsted County, Minnesota, between January 1, 1995, and December 31, 2020. Patients were matched 1 : 1 with controls based on age at diagnosis, sex, smoking status, and body mass index (BMI). Conditional logistic regression was used to examine associations with the odds of EOGC development.

**Results:**

Ninety-six cases of EOGC were identified during the study period. On both univariate and multivariate regression analysis, there was no significant association between use of PPIs, statins, NSAIDs, or metformin and EOGC development. In a final multivariable model, there was a significant reduction in odds of EOGC with COX-2 inhibitor use for six months or more prior to cancer diagnosis (OR = 0.39, 95% CI 0.16-0.94).

**Conclusion:**

In this retrospective, population-based study of individuals in Olmsted County, MN, we found significantly reduced odds of EOGC development associated with COX-2 inhibitor use for six months or more prior to diagnosis, but no association between EOGC development and use of PPIs and other commonly prescribed medications.

## 1. Introduction

Over the past several decades, the incidence of gastric cancer both in the United States and globally has declined [1, 2]. This has been largely due to widespread reduction in traditional risk factors including improved screening and eradication of *H. pylori*, improvements in food processing methods, and public health initiatives to reduce smoking [1]. However, gastric cancer remains a leading cause of cancer death worldwide, with significant morbidity associated with both surgical and medical treatments [1]. Alarmingly, the incidence of early-onset gastric cancer (EOGC) has been increasing in the United States during this same period, now comprising >30% of new gastric cancer cases [2]. The reasons for this trend remain unclear.

Epidemiologic studies show that EOGC is clinically and genetically distinct from traditional gastric cancer and is not associated with standard risk factors such as smoking and obesity [2]. Evidence suggests that chronic acid suppression by PPIs can lead to non-*H. pylori* bacterial overgrowth in the stomach which may cause chronic inflammation and atrophic gastritis [3, 4]. It has also been suggested that a state of hypergastrinemia caused by chronic acid suppression leads to overstimulation of gastric enterochromaffin-like (ECL) cells, potentially leading to tumor development [5]. Present data in the literature are mixed as to the potential contribution of PPI use to gastric cancer. Some studies have demonstrated a significant association between PPI use and gastric cancer development, while others have not found any association [6–9]. Acid suppression with H2-receptor antagonists (H2RAs) has also been evaluated for association with gastric cancer development, and no increased risk has been demonstrated [10].

Other frequently prescribed medications, including statins (HMG-CoA reductase inhibitors), metformin, nonsteroidal anti-inflammatory medications (NSAIDs), and cyclooxygenase-2 (COX-2) inhibitors, have also been associated with a potentially reduced risk of gastric cancer development [11–13]. Statin medications have been proposed to have antiangiogenic, immunomodulatory, and proapoptotic properties based on *in vitro* studies in mice and human gastric cancer cell lines, as well as in retrospective studies [14, 15]. Metformin has been proposed to have a potential protective effect against gastric cancer; however, data are mixed, as some observational studies have demonstrated no significant reduction in gastric cancer risk associated with metformin [11, 16, 17]. NSAIDs and COX-2 inhibitors both exert anti-inflammatory effects thought to potentially protect against gastric carcinogenesis [12, 18]. COX-2 has been associated with gastric carcinogenesis, and increased COX-2 expression in gastric adenocarcinoma has been correlated to depth of tumor invasion and lymph node metastases [19]. In gastric cancer xenograft mice, administration of COX-2 inhibitors has been associated with reduction in tumor size, reduced cancer cell proliferation, and increased apoptosis [20]. In human gastric cancer cell lines, treatment with COX-2 inhibitors has also been associated with cell-cycle arrest and increased apoptosis [21]. However, none of these studies regarding commonly prescribed medications and gastric cancer have focused specifically on EOGC.

Given the global health significance of EOGC and the potential correlation with widely used medications, we sought to conduct a retrospective population-based study to assess whether there is an association between PPI, H2RA, NSAID, COX-2 inhibitor, or metformin use and EOGC development using a population-based medical record linkage system in Olmsted County, Minnesota (MN). This study is the first to our knowledge that specifically focuses on EOGC development risk with regard to these frequently prescribed medications.

## 2. Methods

This study was approved by our local institutional review board (IRB). We used the Rochester Epidemiology Project (REP) database to identify cases of EOGC (defined as development of gastric cancer in patients ≤ 60 years old) in Olmsted County, MN, between January 1, 1995, and December 31, 2020. Age 60 was chosen as the upper age cutoff in this study as prior work has shown an increasing incidence of EOGC over time among this age group, and this work is aimed at elucidating the potential clinical explanation for this [2]. The REP is a comprehensive, validated, medical record linkage system established in 1966 that includes virtually the entire population living in Olmsted County, MN [22–24]. The REP captures all residents of Olmsted County, MN, who have received healthcare services within the county any time after 1966 [24]. Presently, the REP has >6.2 million person-years of follow up on >502,000 unique individuals and has been validated against United States Census data for Olmsted County [24]. Of all individuals eligible for inclusion in the REP, only 2% have requested that their data be removed, making it a validated, comprehensive census of the county's population [24].

We searched the REP utilizing International Classification of Diseases (ICD) diagnosis codes to include all benign and malignant lesions of the stomach and esophagus. Patients with genetic conditions predisposing them to development of EOGC including CDH1 mutation, BRCA mutations, and Lynch syndrome were excluded. Patients with Siewert type II and III gastroesophageal junction (GEJ) tumors were included; those with Siewert type I GEJ tumors were excluded from analysis [25]. Siewert classification was determined through review of the electronic medical record including endoscopy reports, imaging, clinical notes, operative reports, and pathology reports. Gastric cancer diagnosis was confirmed by reviewing pathology reports, imaging, clinical notes, and operative reports. Clinical and demographic data was abstracted from the electronic medical record. Medication use history was considered significant if a patient had been prescribed a given drug for ≥6 months prior to EOGC diagnosis (i.e., the patient should have been taking the drug for six months or more prior to the index diagnosis of gastric cancer). A list of potential healthy controls was generated from the REP database using SAS macros (SAS, Carey, NC), and patients were matched 1 : 1 with controls based on age (within three years), sex, smoking status (current smoker, former smoker, and never smoker), and body mass index (BMI, obese or not obese, with obesity defined as BMI ≥ 30.0). Clinical and demographic data for controls was also abstracted from the electronic medical record.

Descriptive statistics are presented as number (percent) for discrete variables and as median and interquartile range (IQR) for continuous variables; *p* value < 0.05 is considered statistically significant. Conditional logistic regression models, accounting for the 1 : 1 matching of cases with controls, were used to assess the associations between medication use and risk of developing EOGC. Results are presented as odds ratio (OR) and 95% confidence interval (CI), with CI that do not cross 1.0 considered significant. A multiple variable model included as potential covariates all variables of interest, using backward selection to identify the final parsimonious model. A Poisson regression model, using crude incidence rates, was used to examine the association of incidence of EOGC during the study period with age, sex, and year. Incidence rates were adjusted to the US White 2010 census.

## 3. Results

A total of 1552 individual patient records were reviewed from the REP. From these, 96 (6.2%) cases of EOGC were identified and matched 1 : 1 with controls. No patients with genetic syndromes were identified for exclusion. Demographic features of the EOGC patients and matched controls are described in Table 1. Among the EOGC patients, 65 (67.7%) were male, 74 (77.1%) identified as white, and median age at gastric cancer diagnosis was 51 years [43, 55], with a range from 22 to 60 years. Clinical characteristics including medication history for the EOGC cases and controls are described in Table 2.

### 3.1. Power Statement

A total of 96 cases were identified during the study period (January 1, 1995–December 31, 2020). This study would have 80% power to detect a relative change in rates (rate ratio) as small as 2.7. Assuming a linear trend, this would be equivalent to being able to detect an annual rate ratio of 1.05.

### 3.2. Incidence Rate of EOGC over Time

The age- and sex-adjusted incidence for the entire study period from 1995 to 2020 was 5.1 (95% CI 4.0-6.1) per 100,000 person-years (Table 3). The sex-specific age-adjusted rates for females and males were 3.0 (95% CI 2.0-4.1) and 7.2 (95% CI 5.4-8.9), respectively (Table 3). We did not find an overall change in EOGC incidence rate during the study period (Table 3).

### 3.3. Association of Medications with EOGC

PPI use between the cases and controls was similar, with 27 (28.1%) of EOGC patients taking a PPI for at least 6 months at the time of cancer diagnosis vs. 21 (21.8%) of controls (Table 2). PPI use was not significantly associated with an increased risk of EOGC on either univariate (OR 1.38, 95% CI 0.72-2.62) or multivariate (OR 0.58, 95% CI 0.15-2.18) analysis. Additionally, use of H2RAs, statins, NSAIDs, and metformin was also not significantly associated with EOGC risk on either analysis (Table 2). The sole variable that was univariately significant for EOGC development was a history of GERD (OR 1.94, 95% CI 1.06-3.54), but this did not hold on multivariable analysis (OR 2.53, 95% CI 0.78-8.25). In the final parsimonious multivariable model, use of COX-2 inhibitors for ≥6 months at the time of index cancer diagnosis was associated with significantly reduced odds of EOGC development (OR 0.39, 95% CI 0.16-0.94). Significantly increased odds of EOGC development was seen with a history of *H. pylori* infection (OR 8.87, 95% CI 1.03-76.68).

## 4. Discussion

PPIs are important, widely used medications that have drastically improved treatment for GERD, prevention of peptic ulcer disease, and treatment of *H. pylori* [2]. However, the potential long-term effects of chronic acid suppression are not fully understood [3]. In this retrospective, population-based study, we did not find an association between PPI or H2RA use and the development of EOGC. Several other studies have aimed at answering this question [26]. A systematic review of three observational studies demonstrated a dose-dependent association between PPI use and increased gastric cancer risk [6]. Similarly, a retrospective population-based study of over 63,000 patients treated for *H. pylori* demonstrated a significant association with PPI use and gastric cancer risk that paralleled the frequency and duration of PPI use [7]. However, a causal link has not yet been established, and other studies have failed to show an association between PPIs and the development of precancerous gastric lesions, including a large multicenter, double-blind clinical trial at over 500 centers in 33 countries [27].

Other commonly prescribed medications have been proposed as possibly chemopreventive for gastric cancer development, including statins, NSAIDs, metformin, and COX-2 inhibitors [11–14]. However, existing studies have not yet specifically examined the potential protective role of these medications in EOGC. In this patient cohort, we found a significant reduction in EOGC risk in patients who had been taking COX-2 inhibitors for six months or more prior to index gastric cancer diagnosis. This is consistent with what has been reported elsewhere in the literature and with the anticarcinogenic effects of COX-2 inhibitors demonstrated *in vitro* [19–21]. These medications present a potential target for gastric cancer chemoprevention, but are not risk-free. As such, future, high-quality studies are needed to better elucidate this association.

We did not observe a protective effect of statins or metformin against EOGC, which differs from other published studies [11]. This may be due to the fact that low numbers of EOGC patients and controls were taking these medications in our cohort. This group may not have had adequate power to detect a difference with use of these medications. Additionally, we did not observe an association between EOGC and NSAIDs, which have been previously shown to have a potential protective effect against gastric cancer development [12]. This is also potentially due to the low number of patients in our cohort taking these medications, with actually the same number of patients reported to be taking NSAIDs in each group. Assessing the impact of NSAIDs on EOGC development in this cohort was also likely limited by the fact that patients may have been taking these medications intermittently on an as needed basis, and this was not well reflected in the medical record.

We did not find an overall increase in the incidence rate of EOGC during the study period. One factor that may have contributed to this is that the vast majority of patients in this cohort are Caucasian, due to the existing demographics in Olmsted County, MN. Prior work that observed an increase in incidence rates of EOGC over time had a substantially more diverse study population, and the greatest observed incidence rates of EOGC were in nonwhite patients [2]. As such, our findings are likely only applicable to a Caucasian population, and we are unable to comment on how EOGC rates may vary in a more diverse population.

This study has additional limitations. Using a combination of the REP and the electronic medical record, we were able to obtain prescription information from medication lists and clinical notes, but we are unable to verify whether patients were actually taking medications as prescribed, which may also be impacting our results. Additionally, the duration of medication use varied among patients and controls, but given the small sample size and limitations of specific data available through the REP, we were unable to meaningfully comment on how duration of use may impact EOGC risk. We did not identify any cases of inherited gastric cancer syndromes in this cohort. However, it may be that some of these cases were unrecognized as genetic in our review due to limited clinical documentation.

There is presently no clear explanation for why the rates of EOGC have increased, despite an overall decrease in gastric cancer incidence globally over the last several decades [2]. Genetic syndromes including CDH1 mutation are well known to cause EOGC, but the frequency of these mutations has not changed, pointing to alternate environmental causes. In addition to medication use, viral infection is another potential etiology for this trend. A recent multicenter study found that rates of Epstein-Barr virus (EBV) subtype gastric cancer were significantly greater in younger patients (≤45 years) compared to older patients (≥55 years). In fact, 15 of the 17 EBV-associated gastric cancer cases in that study were in patients less than 68 years old, which is the median age of gastric cancer diagnosis in the United States [28]. Further investigation into alternate environmental causes including potential infectious etiologies will be needed to elucidate this epidemiologic trend.

## 5. Conclusion

In this retrospective, population-based study of individuals in Olmsted County, MN, we were not able to identify an association between several commonly implicated medications including PPI, H2RA, metformin, NSAID, and statin use and risk of EOGC development. We did observe a significant reduction in EOGC risk with the use of COX-2 inhibitors. EOGC remains a substantial source of morbidity and mortality for many patients globally, and the cause for the increasing incidence of this disease is still unknown. This is the first study to our knowledge that specifically looks at the association of EOGC with commonly prescribed medications. Additional studies will be needed to better assess the potential environmental causes of EOGC.

## Figures and Tables

**Table 1:** Demographic characteristics of the EOGC patient and control patient groups.

	EOGC patients (*n* = 96)	Control patients (*n* = 96)
Age at cancer diagnosis (years)	51 [43, 55]	—
Sex (% male)	65 (67.7%)	65 (67.7%)
Racial demographics		
White	74 (77.1%)	78 (81.2%)
Black	6 (6.2%)	5 (5.2%)
Hispanic/Latino	3 (3.1%)	1 (1.0%)
Asian	4 (4.1%)	2 (2.1%)
Other	3 (3.1%)	1 (1.0%)
Unknown	6 (6.2%)	9 (9.3%)

Age described as median with [interquartile range]. Patient-reported race from the electronic medical record was utilized. Patients with “unknown” race are those who did not have a race reported in the medical record. EOGC = early-onset gastric cancer, defined as index cancer diagnosis at age ≤ 60 years old. Control patients were matched 1 : 1 with EOGC patients based on age (within three years), sex, smoking status (current smoker, former smoker, and never smoker), and BMI = body mass index (obese or not obese, with obesity defined as BMI ≥ 30.0).

**Table 2:** Clinical characteristics of EOGC patient and control patient groups.

	EOGC patients (*n* = 96)	Control patients (*n* = 96)	Univariate odds ratio estimate (95% confidence interval)	Multivariate odds ratio estimate (95% confidence interval)
PPI use	27 (28.1%)	21 (21.8%)	1.38 (0.72-2.62)	0.58 (0.15-2.18)
H2RA use	8 (8.3%)	2 (2.1%)	4.0 (0.85-18.84)	2.24 (0.427-11.8)
BMI (kg/m^2^)	29.2 [25.0, 34.2]	29.5 [26.9, 34.3]	—	—
Smoking status at time of diagnosis			—	—
Never	44 (45.8%)	43 (44.7%)		
Former	24 (25.0%)	34 (35.4%)		
Current	28 (29.1%)	19 (19.8%)		
History of *H. pylori* infection	7 (7.2%)	2 (2.1%)	3.50 (0.73-16.85)	**8.87 (1.03-76.67)**
History of GERD	42 (43.8%)	27 (28.1%)	**1.94 (1.06-3.54)**	2.53 (0.78-8.25)
Prior stomach surgery	6 (6.2%)	0	13.85 (0.612-313.35)	9.35 (0.41-213.22)
Metformin use	4 (4.2%)	9 (9.4%)	0.44 (0.14-1.44)	0.54 (0.15-1.99)
NSAID use	23 (24.0%)	23 (24.0%)	1.00 (0.52-1.92)	—^∗^
COX-2 inhibitor use	12 (12.5%)	22 (22.9%)	0.44 (0.19-1.02)	**0.39 (0.16-0.94)**
Statin use	15 (15.6%)	14 (14.6%)	1.1 (0.47-2.59)	—^∗^

^∗^Not included in the final parsimonious multivariable model due to *p* value of >0.8 on univariate analysis of maximum likelihood estimates. Odds ratio estimates with 95% confidence intervals that do not cross 1.0 considered significant (bolded). BMI = body mass index, described as median with [interquartile range]. Discrete variables expressed as counts (percent). PPI = proton-pump inhibitor; H2RA = H2-receptor antagonist; GERD = gastroesophageal reflux disease; NSAID = nonsteroidal anti-inflammatory agent; COX-2 inhibitor = cyclooxygenase-2 inhibitor; statin = HMG-CoA reductase inhibitor. EOGC = early-onset gastric cancer, defined as index cancer diagnosis at age ≤ 60 years old. Control patients were matched 1 : 1 with EOGC patients based on age (within three years), sex, smoking status (current smoker, former smoker, and never smoker), and BMI (obese or not obese, with obesity defined as BMI ≥ 30.0). Patient was considered to have a history of use for each medication if they had been prescribed the medication for six months or more prior to index diagnosis of gastric cancer.

**Table 3:** Incidence rates of EOGC in Olmsted County, MN, from 1995 to 2020.

	1995-2020	1995-1999	2000-2004	2005-2009	2010-2014	2015-2020
Age-adjusted female	3.0 (2.0-4.1)	2.2 (0.0-4.8)	3.2 (0.6-5.7)	4.5 (1.6-7.5)	2.7 (0.5-4.8)	2.7 (0.6-4.7)
Age-adjusted male	7.2 (5.4-8.9)	9.3 (4.0-14.5)	6.6 (2.4-10.8)	7.2 (3.2-11.1)	9.0 (4.9-13.2)	4.6 (1.9-7.4)
Age- and sex-adjusted	5.1 (4.0-6.1)	5.7 (2.8-8.6)	4.9 (2.4-7.3)	5.8 (3.4-8.3)	5.8 (3.5-8.2)	3.6 (1.9-5.3)

Age-adjusted sex-specific and age- and sex-specific rates of EOGC in Olmsted County, MN, calculated using Poisson regression, adjusting population US White 2010 census. EOGC = early-onset gastric cancer, defined as index cancer diagnosis at age ≤ 60 years old.

## Data Availability

Deidentified data utilized in this study can be obtained upon reasonable request to Dr. Travis E. Grotz (grotz.travis@mayo.edu).
